# Supplementary material for: Ocean-bottom seismometers reveal surge dynamics in Earth’s longest-runout sediment flows
Source: Commun Earth Environ. 2025 Feb 25;6(1):147. doi: 10.1038/s43247-025-02137-z (PMC11850286; doi:10.1038/s43247-025-02137-z)
Supplement: Supplementary file 2 — Supplementary Information [file 43247_2025_2137_MOESM2_ESM.pdf]

Supplementary Information for

Ocean-bottom seismometers reveal surge dynamics in Earth's longest-runout sediment flows

Pascal Kunath[1*], Peter J. Talling[2,3], Dietrich Lange[1], Wu-Cheng Chi[4], Megan L. Baker[3], Morelia Urlaub[1,5], Christian Berndt[1]

[1]GEOMAR Helmholtz Centre for Ocean Research; Wischhofstraße 1-3, 24148 Kiel, Germany.

[2]Department of Earth Sciences, Durham University; South Road, Durham, DH1 3LE, UK.

[3]Department of Geography, Durham University; South Road, Durham, DH1 3LE, UK.

[4]Institute of Earth Sciences, Academia Sinica; 128, Sec. 2, Academia Road, Taipei 11529, Taiwan

[5]Kiel University, Christian-Albrechts-Platz 4, 24118 Kiel, Germany

Corresponding author: Pascal Kunath (pkunath@geomar.de)

Content of this file:

Figures S1 – S7

Supplementary Notes1

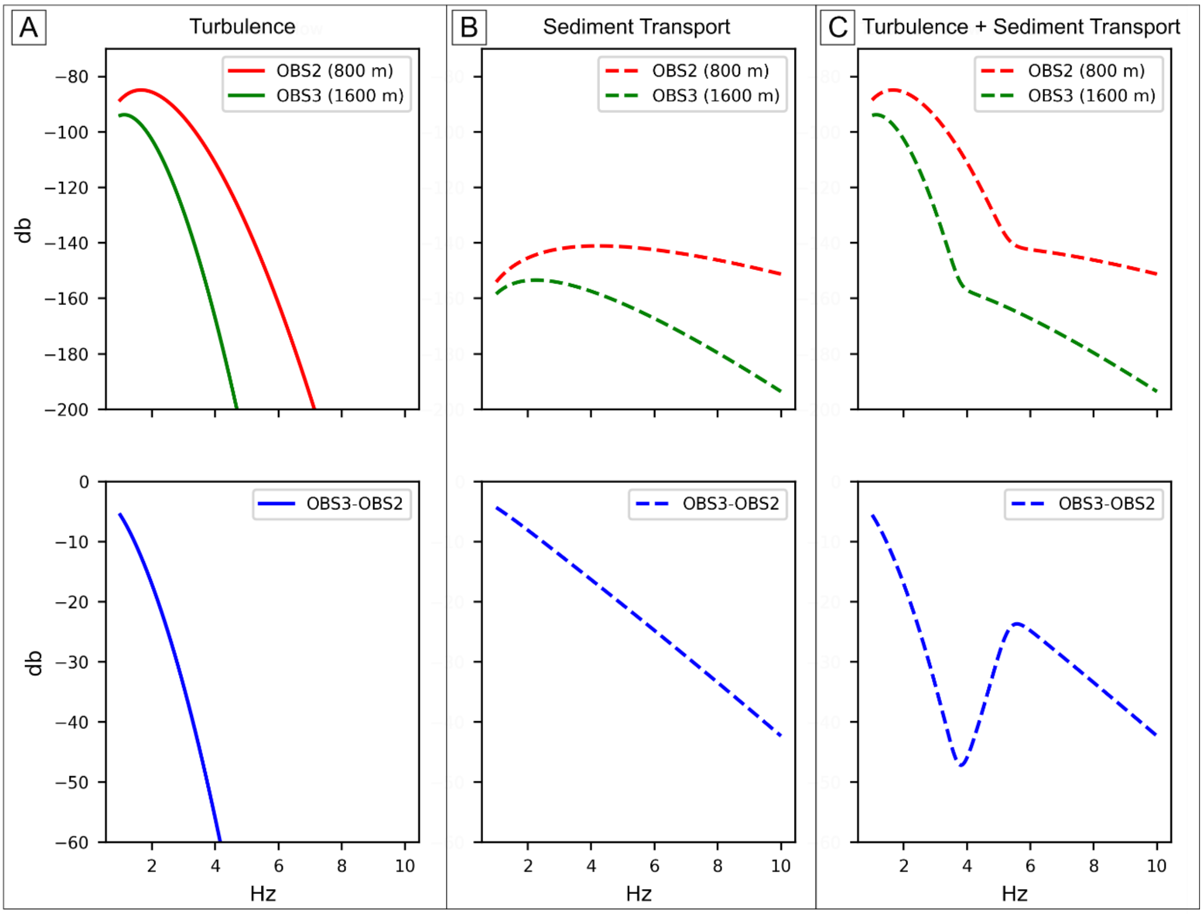

Figure S1. PSD generated by flow turbulence and bedload transport based on models from Gimbert et al.[30] and Tsai et al.[31]. Modelling uses flow parameters listed in Table 1. (A to C upper panel) Seismic spectra for two stations located 800m (red dotted line) and 1600m (green dotted line) from the flow, showing the spectra for (A) flow turbulence, (B) sediment transport, and (C) a combination of flow-turbulence and sediment transport. These distances from the flow are comparable to those of OBS2 and OBS3 in the Congo Canyon (Fig. 1). (A to C lower panel) Frequency spectra showing the difference in strength between seismic signals measured at 800 m and 1600 m from the flow, obtained by subtracting measurements at 1600 m from those at 800 m. When either water turbulence or sediment transport acts as the sole process, the seismic power decreases monotonically with frequency, though the rate of decrease differs between the two processes. However, when both processes are present, and their spectral contributions overlap, as shown in (C), the resulting discrepancy creates a non-monotonic notch-like pattern in the spectra.

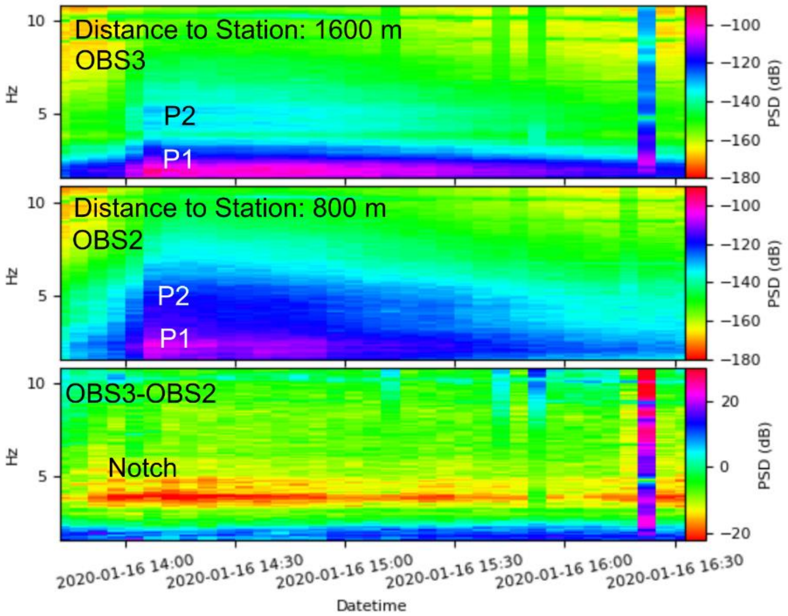

Figure S2. Spectrograms for the January 16th turbidity current recorded at (upper panel) OBS3 and (middle panel) OBS2, and their (lower panel) difference. The OBS stations are located in close proximity, within < 3 km distance from each other, but on opposite sides of the canyon (Fig. 1b for location). A comparison of the turbidity spectral signatures from OBS2 and OBS3 reveals two distinct phases: one at 1-3 Hz (P1) and another at 4-6 Hz (P2). At the larger distance, seismic power is concentrated in the 1-3 Hz phase, with the 4-6 Hz phase attenuated (upper panel). In contrast, both phases show strong power at the shorter distance (middle panel). The difference between the spectral signatures from both stations shows a non-monotonic behavior, with a distinct notch around 3-5 Hz (lower panel).

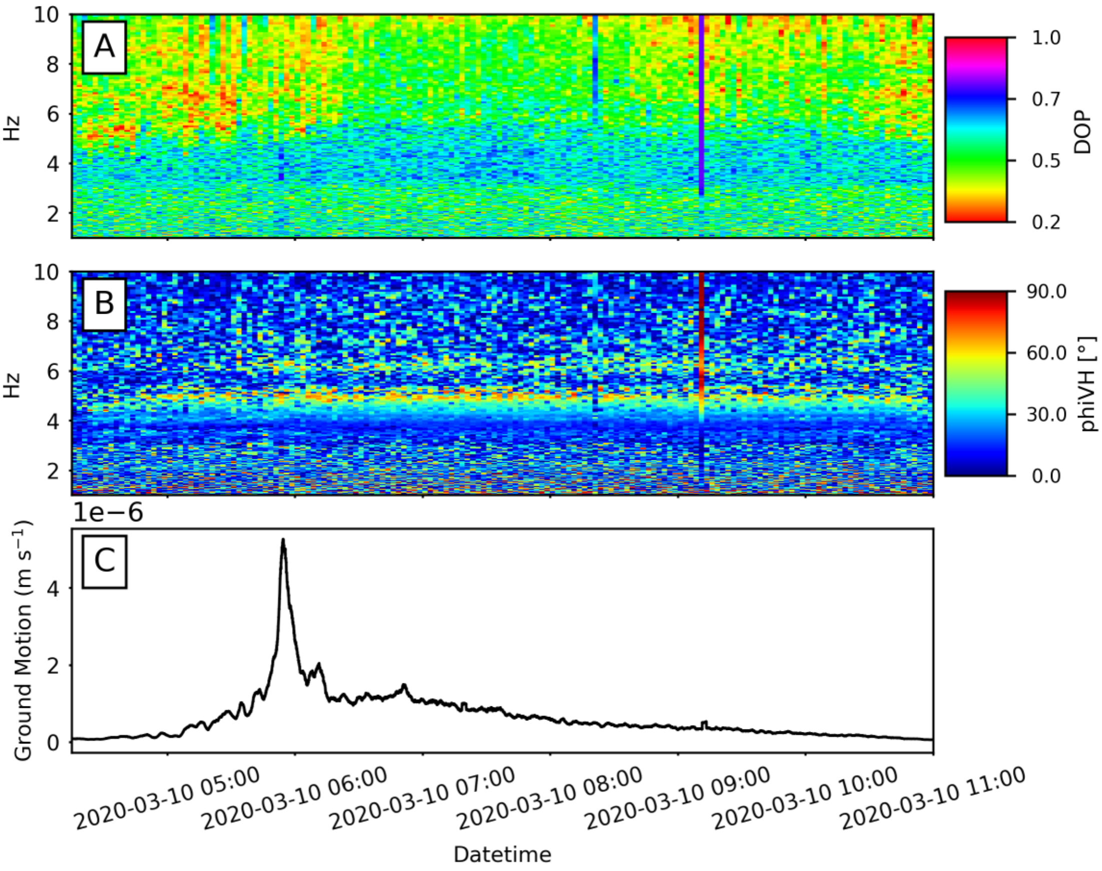

Figure S3. Frequency-dependent polarization analysis of the March 8th event at OBS6. (A) Displays the Degree of Polarization (DOP) as a function of time and frequency. A higher DOP indicates greater stability in the signal's polarization within the specified time-frequency window, enhancing the reliability and accuracy of Scholte wave derived back azimuth estimations. The turbidity current signal demonstrates high polarization between 3 – 5.5 Hz. (B) Shows the magnitude of the vertical-horizontal phase angle (phivh) over time and frequency. Notably, phivh approximates 90° for frequencies between 4-6 Hz during the turbidity current flow, which in combination with the high degree of polarization is indicative of locatable Scholte-waves and their respective sources. (C) Presents the envelope of vertical component turbidity current seismic waveform.

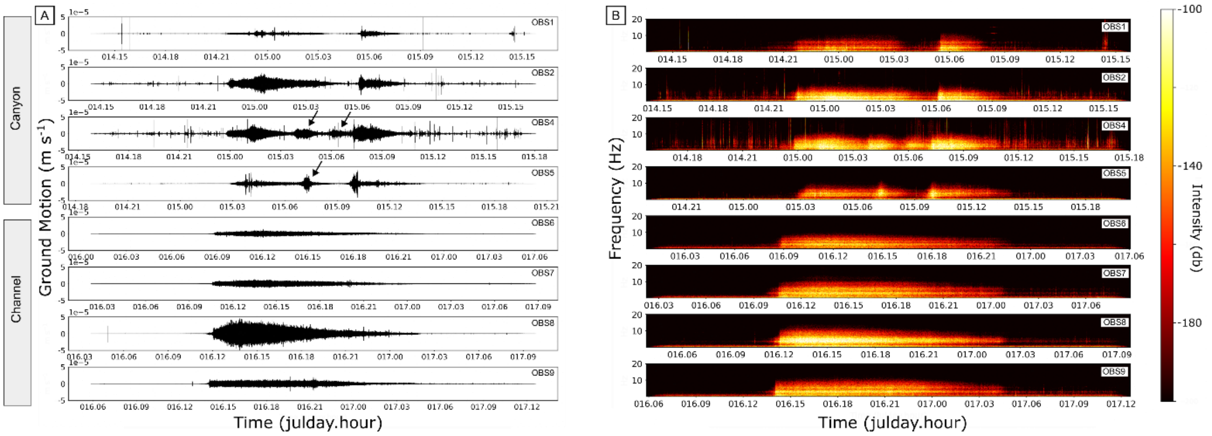

Figure S4. The seismic signals from the January 14, 2020 Congo Canyon turbidity current were recorded by OBSs at multiple locations along the flow path, ranging from OBS 1 to OBS *9* (see figure 1 for locations). The subpanels are arranged in order of distance from the coastline. The OBS signals reveal spatial-temporal variations in the turbidity current flow. Notably, multiple pulses are observed at shallower water sites (black arrows), which amalgamate before reaching deeper water sites. (B). The corresponding spectrograms of the waveforms presented in section A show that the OBS signals exhibit strong signals centered around the 1-6 Hz frequency range.

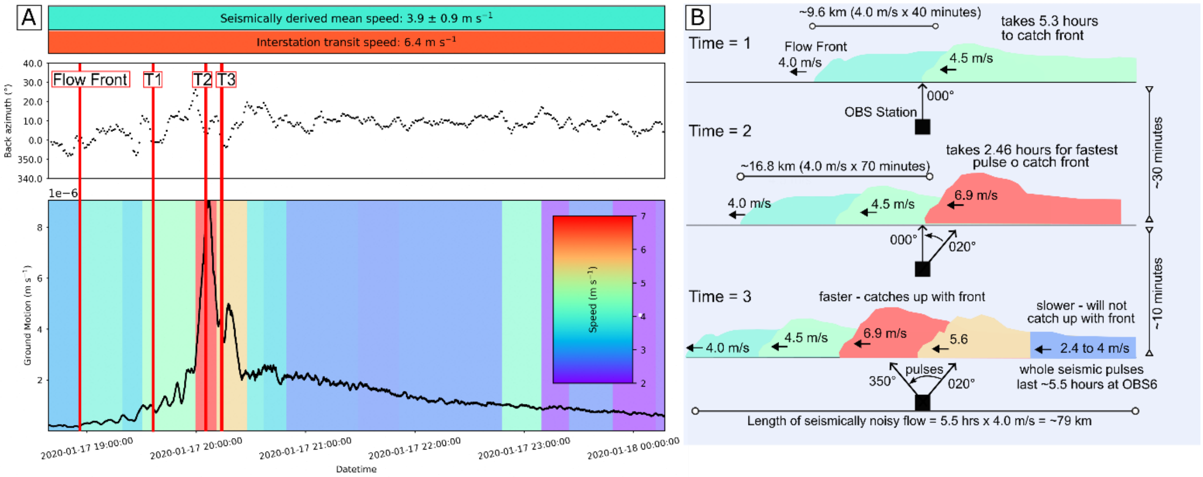

Figure S5. Analysis of seismic signals from the canyon-flushing turbidity current on January 16, 2020, recorded at OBS 6 near the Congo Submarine Channel. (A) *Upper panel*: The time series of back-azimuth (BAZ) directions indicates multiple surges moving down the channel. For each surge, the BAZ shifts from upstream directions (10° and above) as it approaches OBS 6, to downstream directions (decreasing below 0°) as the surge moves away from the station. Vertical red lines (Time = 1 to 3) mark specific instances corresponding to snapshots of the flow structure shown in (B). *Lower panel*: Vertical component ground motions of the seismic waveforms recorded at OBS 6 for the January 16, 2020, turbidity current, with the derived speed structure displayed in the background. The interstation transit speed and seismically derived mean speed are plotted as a bar above. The speed estimate from the fastest part of the flow aligns with the interstation transit speeds. However, average speeds are slower due to the contribution of the slower-moving trailing part behind the fastest and most seismically noisy pulses, which constitute approximately two-thirds of the overall signal duration. (B) Snapshots of the flow based on observations in (A). The flow front is approximately 40% slower compared to the fastest and most seismically noisy surge, which passes the station 70 minutes later—corresponding to a trailing distance of approximately ~17 km, assuming constant frontal speeds.

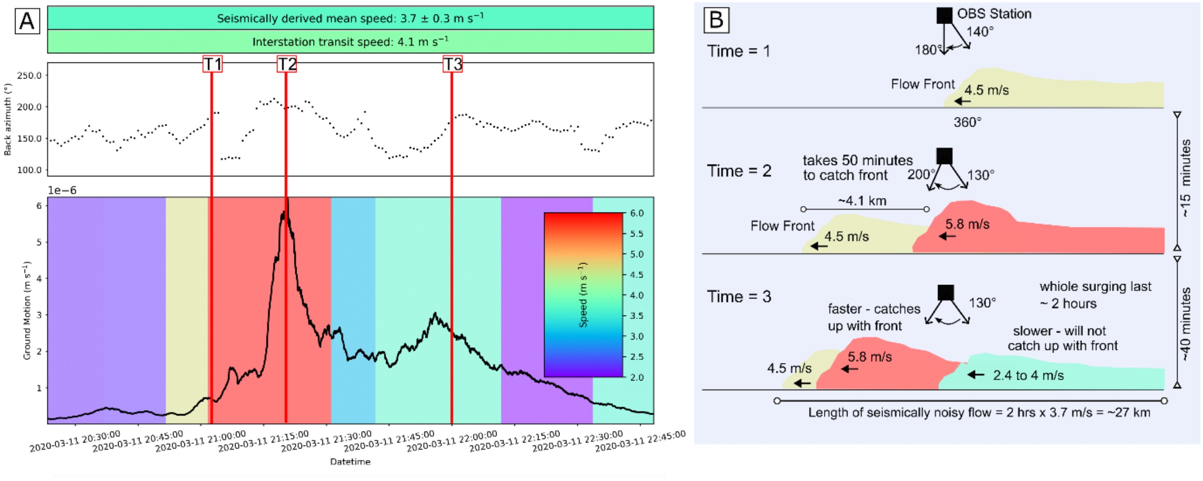

Figure S6. Analysis of seismic signals from the March 11, 2020 turbidity current, recorded at OBS 4 near the Congo Submarine Canyon. (A) Upper panel: The back-azimuth (BAZ) time series reveals multiple surges moving down the canyon. As each surge approaches OBS 4, the BAZ shifts from upstream (130° - 180°) to downstream (180° - 200°) as the surge passes and moves away. Vertical red lines (Time 1 to 3) indicate specific moments corresponding to the flow snapshots in (B). Lower panel: Vertical component seismic waveforms recorded at OBS 4 during the event, with the speed structure overlaid in the background. Interstation transit speed and seismically derived mean speed are represented as a bar above, showing alignment between speed estimates and transit speeds. (B) Flow snapshots based on the observations in (A). The leading edge of the flow is approximately 22% slower than the fastest, most seismically noisy trailing surge, which passes OBS 4 about 15 minutes later—corresponding to a trailing distance of ~4.1 km, assuming constant frontal speeds.

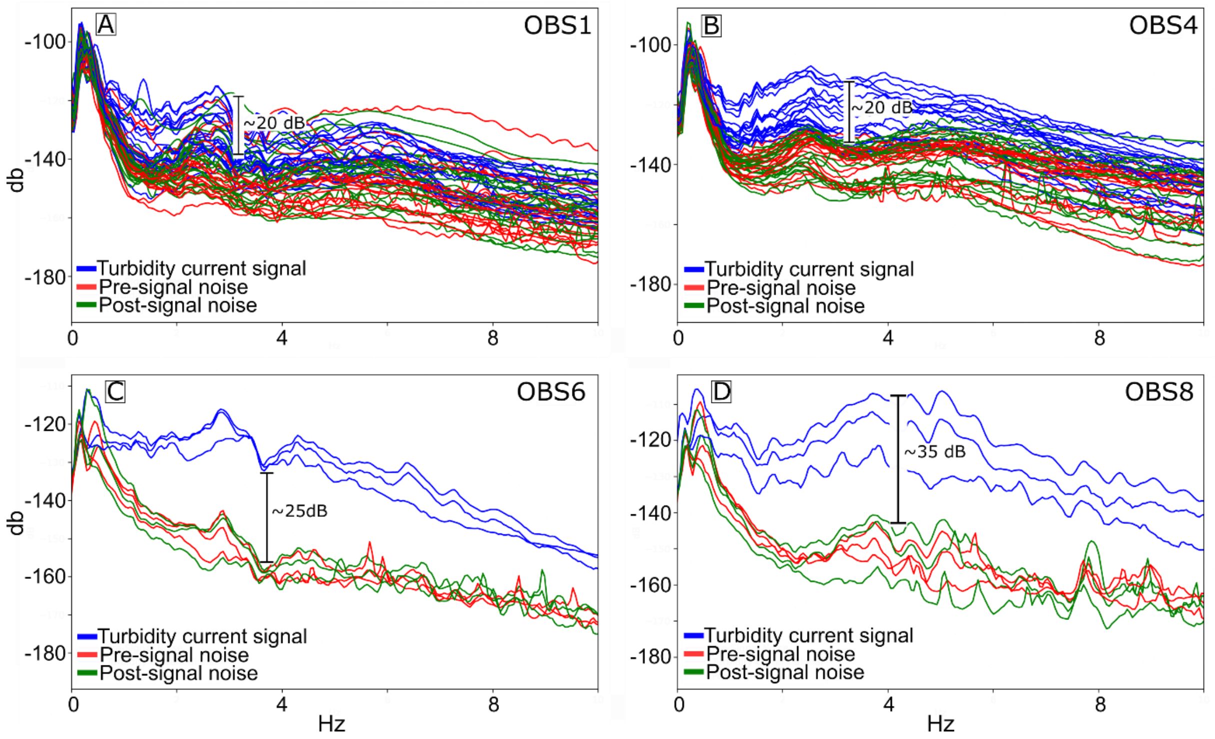

Figure S7. Power spectral densities from all twenty turbidity current events detected during the deployment period from October 2019 to May 2020, including their respective pre- and post-signal noise levels, from stations located in the upper canyon (OBS1 (A) and OBS4 (B)) and the deep-water channel (OBS6 (C) and OBS8 (D)) (refer to Figure 1 for locations). The signal-to-noise ratios for these seismic events ranged from 5 to 30 dB above ambient noise levels. Notably, lower signal-to-noise ratios were predominantly recorded in the upper canyon region, which is affected by seismic noise from shallow water sources and anthropogenic sources, such as shipping and drilling activities, that also contribute to high-frequency noise.

Supplementary Notes 1: Tracking the source region using the back azimuth estimates from polarization analysis

Our results demonstrate the capability of seismology to revolutionize turbidity current monitoring by documenting the finer-scale spatiotemporal evolution of these powerful flows. However, there are challenges and lessons learned that we encountered.

First, we encountered challenges in accurately estimating the back azimuth (BAZ) of the turbidity current seismic sources, particularly in areas where the canyon meanders close to the OBS station. The primary difficulty arose from the inherent 180-degree ambiguity in deriving BAZ using single-station polarity and particle motion analysis. This limitation meant that the flow could be on either side of the station in meandering sections, with signals potentially radiating from various locations within the flow and along the channel, especially when larger currents emit signals over significant distances. As a result, the BAZ evolution, influenced by moving line sources, could lead to near-constant or slowly adjusting BAZ values or scattering effects from multiple prominent locations within complex canyon geometries as the flow progresses downslope. In either case, these patterns are very difficult to interpret without additional constraints, such as ADCP measurements.

In these cases, the leading edge of the turbidity current flow is where the highest confidence in back azimuth estimates—and thus source location and velocity—is achieved. This is because these segments radiate within a relatively narrow back azimuth range due to their greater distance from the station, which reduces the sensitivity to variations in the source location. Consequently, the seismic waves from these segments exhibit more consistent directionality when approaching the station.

Interestingly, we did not observe precise back-azimuth estimates at the end of some flows that would typically indicate a down-canyon moving source. This may be because the flow extends beyond the detection sensitivity of the OBS as it becomes more dilute, no longer generating the distinctive, highly polarized Scholte waves required for accurate analysis. Nonetheless, the flow could still influence the derived back azimuth (BAZ), albeit with reduced precision.

Second, we estimated source locations by projecting the derived back azimuth onto the line of steepest descent along the channel axis, assuming the flow is confined within it. However, given that the canyon's width can vary between 500 to 2000 meters along the entire canyon-channel system, this introduces uncertainty—especially in meandering sections where seismic sources may actually be located several hundred meters off this line, and potentially even more in cases of overbank flow.

Optimizing seismic station placement can reduce uncertainty and enhance detection capabilities. As demonstrated with OBS6, in simpler, nearly straight channel geometries, both the location uncertainty and the 180-degree ambiguity are minimized, yielding more accurate results. By combining data from several stations and positioning seismometers closer to achieve overlapping near-field detection ranges, it might be possible to achieve continuous capture of the seismic signal. This would provide more robust

information on flow location, velocity, cross-sectional area, event sequencing, and dynamics. In addition, deploying a small-aperture OBS array could help to obtain more information about Scholte waves by providing crucial slowness and incident angle information of incoming rays. With at least three stations registering a turbidity current signal, the seismic source location could be determined in a time-resolved manner using multiple independent techniques[29]. This approach enables more confident constraints on flow propagation speed, particularly in complex channel geometries where assumptions of flow confinement are less reliable.
